# “A Time to Tear Down and a Time to Mend”: The Role of Eicosanoids in Atherosclerosis

**DOI:** 10.1161/ATVBAHA.124.319570

**Published:** 2024-10-24

**Authors:** Samuel C.R. Sherratt, R. Preston Mason, Peter Libby, Deepak L. Bhatt

**Affiliations:** Mount Sinai Fuster Heart Hospital, Icahn School of Medicine at Mount Sinai, New York, NY (S.C.R.S., D.L.B.).; Elucida Research, Beverly, MA (S.C.R.S., R.P.M.).; Department of Medicine, Cardiovascular Division, Brigham and Women’s Hospital, Harvard Medical School, Boston, MA (R.P.M., P.L.).

**Keywords:** atherosclerosis, eicosanoids, fatty acids, omega-3, fatty acids, omega-6, inflammation

The wisdom of Solomon manifests throughout the natural world, in which the cycle of destruction and rebuilding sustains life and health. For example, the body’s response to pathogens or acute tissue injury sets in motion the pathophysiologic process of inflammation. In precise fashion, the activated innate immune system triggers resident immune cells (eg, CCR2^−^ [C-C motif chemokine receptor 2] macrophages) to recruit circulating leukocytes to the inflamed tissue (neutrophils are generally the first responders) and create a pro-inflammatory environment to neutralize microbial invaders or clear debris. These same cells, joined by CCR2^+^ macrophages, then mediate the conversion to an anti-inflammatory and pro-resolution state to restore tissue homeostasis. The timing and extent of this inflammatory process (and its resolution) involves a series of complex fatty acid metabolites derived from polyunsaturated fatty acids (PUFAs). These PUFAs reside in cellular phospholipids where they modulate membrane structure, protein function, and lipid raft organization.^1^ These same PUFAs furnish substrate for synthesis of bioactive lipid metabolites, which act as secondary messengers with direct and potent biological actions. While multiple chemical classes of these mediators exist, the oxygenated metabolites, often termed oxylipins, possess the widest array of cellular actions. These metabolites are generated enzymatically by tissue-specific lipoxygenases (LOXs), cyclooxygenases (COXs), and cytochrome P450 (CYP; Figure 1).

**Figure 1.**
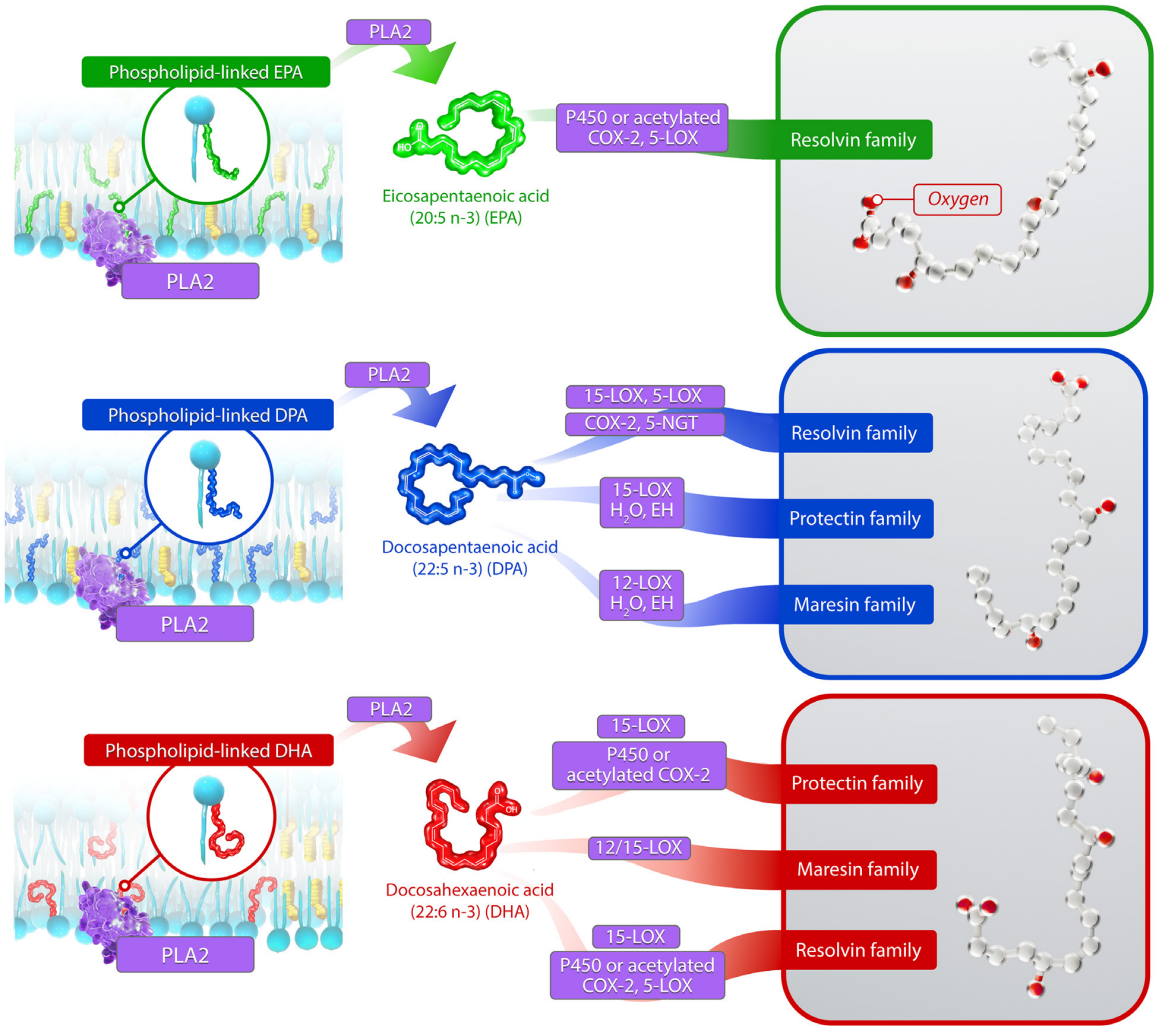
**Multiple enzymes, including COXs (cyclooxygenases), LOXs (lipoxygenases), and CYP (cytochrome P450), generate pro-resolving oxylipins from omega-3 fatty acid (n3-FA) precursors.** The n3-FAs concentrate in cell membranes and are then released enzymatically by PLA_2_ (phospholipase A_2_) before conversion to pro-resolving oxylipins by P450, 12/15-LOX, and COX2 (including acetylated COX2). The structures of representative oxylipins from each n3-FA are shown in the boxes on the **right**, where the oxygen atoms are highlighted red. 5-NGT indicates 5-nitrosoglutathione; DHA, docosahexaenoic acid; DPA, docosapentaenoic acid; EH, epoxide hydrolase; and EPA, eicosapentaenoic acid.

## INTERPLAY BETWEEN OXYLIPINS AND INFLAMMATION

In atherosclerotic cardiovascular disease (ASCVD), oxylipins play a concerted role in vascular inflammation where they serve both to initiate and then resolve inflammatory processes under the control of various cellular mediators.^2^ Under homeostatic conditions, sufficient oxylipins are available to temporally support each stage of inflammation in the arterial wall. However, there can develop an imbalance in PUFA substrate concomitant with an increase of other pro-inflammatory stimuli, most notably the cytokines and cellular debris. In the case of atherosclerosis, macrophage phagocytosis of retained ApoB-containing lipoproteins and dead or damaged cells in the intima can lead to chronic, smoldering inflammation, culminating in atheroma evolution.^3,4^ In the case of myocardial infarction, or other forms of tissue injury, damage associated molecular patterns derived from cellular debris provoke inflammation by ligating pattern recognition receptors (eg, the toll-like receptors). Therefore, there is active pursuit of therapeutic strategies aimed at optimizing the integrated actions of oxylipins in homeostasis to reduce ASCVD risk and ischemic events.

Arguably, the most consequential and well-characterized lipid ratio for determining inflammatory mediator flux is that of omega-6 fatty acids (n6-FA) to omega-3 fatty acids (n3-FA). The ratio of n6-FA to n3-FA in the Western diet has increased from ≈4:1 to over 20:1 during the past century due to widespread introduction of highly processed oils and foods.^5^ The key representatives of each class are arachidonic acid (AA; 20:4 n6) and eicosapentaenoic acid (EPA; 20:5 n3), respectively, and the ratio of EPA/AA is a well-established marker of ASCVD risk.^6,7^ Both fatty acids have 20 carbon atoms in their acyl chains and a diverse array of metabolites collectively referred to as eicosanoids. The diet provides the primary source of EPA and AA (as well as other long-chain PUFAs), as the conversion of the shorter PUFA species α-linolenic acid (18:3 n3) and linoleic acid (18:2 n6) to EPA and AA, respectively, is inefficient in men and postmenopausal women. Thus, direct intake of these PUFAs via diet is the most effective means of achieving adequate levels. While PUFAs circulate within multiple lipid environments, including lipoproteins and red blood cells, and have important biological actions,^8^ peripheral tissues will take up EPA and AA and incorporate them into cellular phospholipids as needed in response to physiological requirements.^9^ Importantly, EPA and AA will compete for binding at the *sn2* position of phospholipids, a key consideration for determination of the EPA/AA ratio.

In the membrane, these PUFAs differentially modulate membrane fluidity, width, and cholesterol localization.^1^ The EPA molecule has an extended, stable conformation that maintains favorable membrane cholesterol distribution and lipid dynamics while facilitating effective free radical trapping. These physicochemical properties of EPA reduce the formation of membrane cholesterol domains that precipitate extracellular crystals as compared with other PUFAs.^1^ Phospholipase A_2_ can then hydrolyze the acyl chains of these phospholipids and provide substrate to intracellular enzymes for oxylipin production. In addition to their competition for phospholipid incorporation, EPA and AA compete for COX1 and COX2 binding to differing degrees, with EPA reducing AA oxygenation by COX1 more effectively than by COX2.^10,11^ Thus, the EPA/AA ratio not only functions to normalize circulating EPA levels but also reflects the balance between n3-FA and n6-FA actions in target tissues, from the membrane to the oxylipins produced by those same tissues.

## ROLE OF EICOSANOIDS IN VASCULAR INFLAMMATION

The eicosanoids derived from EPA and AA have critical roles at each stage of the vascular inflammatory process.^2,12^ When inflammatory signals are released, leukotriene B_4_, which is an AA-derived 5-LOX product, triggers infiltration of polymorphonuclear neutrophils (PMNs) to atherosclerotic lesions or other diseased tissues. COX-dependent production of prostaglandin E_2_ from AA facilitates this recruitment by vasodilation to boost trafficking PMNs to the emerging lesion. Following the first wave of debris clearance by these PMNs, there is an important switch in the eicosanoid class away from pro-inflammatory to pro-resolving. Prostaglandin E_2_, which initially served to stimulate the inflammatory cascade, now induces 15-LOX within PMNs to produce lipoxin A_4_.^13^ This switch both limits leukotriene B_4_ production to reduce further PMN infiltration and stimulates clearance by macrophages of dead cells (efferocytosis).

Oxylipins from the n3-FAs EPA, docosahexaenoic acid (DHA), and docosapentaenoic acid also increase in production to promote further inflammation resolution. These oxylipins are collectively referred to as specialized pro-resolving mediators (SPMs) and bind to specific G-protein–coupled receptors to facilitate intracellular actions. SPMs fall into 3 categories, based on their lipid precursor and cellular actions: resolvins, formed from EPA, DHA, and docosapentaenoic acid, and protectins and maresins, both of which are only formed from DHA and docosapentaenoic acid.^14^ Representative oxylipins from these n3-FA precursor possess antiatherosclerotic properties. For example, resolvin E1 (RvE1), which is formed from EPA by successive conversion to 18-hydroxyeicosapenatenoic acid via CYP and acetylated COX2 and then RvE1 via 5-LOX in PMNs, reduced lesion size and inflammatory biomarker levels in multiple animal models of atherosclerosis.^15,16^ EPA and EPA-derived oxylipins including RvE1 localized preferentially to thin-cap atheromas in *Apoe*^*–/–*^ mice.^17^ 18-hydroxyeicosapenatenoic acid itself also prevents diapedesis of PMNs.^18^ Resolvin D1, formed from DHA by way of a 17-hydroperoxy-DHA intermediate by 15-LOX, also reduced lesional leukotriene B_4_ levels, necrotic core size, and oxidative stress in *Ldlr*^*–/–*^ mice.^19^

The in vivo concentrations of SPMs in humans and hence their clinical relevance has engendered considerable debate, as different laboratories use a wide range of methods for determining quantitation limits in the LC-MS/MS (liquid chromatography-tandem mass spectrometry) platforms.^20–22^ While some studies have failed to detect SPMs in patients, many other studies have shown levels of various SPMs are modulated with n3-FA treatment.^22^ Specific to EPA-only formulations, clinical studies have shown that treatment leads to increases in E-series resolvins, as well as 18-hydroxyeicosapenatenoic acid in a dose- and time-dependent manner.^23,24^ Analysis of SPMs in blood samples from large cardiovascular outcome trials using 4 g/d icosapent ethyl (IPE) would further clarify their clinical relevance. There appears little question that SPMs can elicit biological actions, and these actions by n3-FA oxylipins underscore the importance of supplying the organism (through diet and intervention) with a sufficient balance of n6-FA and n3-FA to facilitate a self-resolving arc of the inflammatory response.

## DISTINCT CARDIOVASCULAR BENEFITS OF ICOSAPENT ETHYL

However, contemporary clinical trials investigating n3-FA formulations for both secondary and primary prevention of cardiovascular events have, until recently, generally shown that fish oil supplementation has no benefit.^25^ Whether these findings were due to improved standard of care regimens (eg, statins), the patient populations, the dose of n3-FA administered, or the n3-FA composition remains unsettled. But in the case of ASCVD, in 2019, REDUCE-IT (Reduction of Cardiovascular Events With Icosapent Ethyl—Intervention Trial) showed that administration of 4 g/d IPE to high-risk, statin-treated patients with established hypertriglyceridemia reduced major adverse cardiovascular events by 25%.^26^ IPE is a highly purified ethyl ester of EPA. Despite significant reductions in triglyceride levels, the biomarker found to be most predictive of event reduction and mediate the most benefit was the serum EPA level.^27,28^ These results were supported by the only other trial of n3-FAs to yield a positive result before REDUCE-IT, the open-label JELIS trial (Japan EPA Lipid Intervention Study). Here, plasma levels of EPA again correlated inversely with major coronary events in statin-treated patients receiving 1.8 g/d IPE.^29^

Most recently, the RESPECT EPA trial (Randomized Trial for Evaluation in Secondary Prevention Efficacy of Combination Therapy–Statin and Eicosapentaenoic Acid) investigated the effects of 1.8 g/d IPE in Japanese patients with documented coronary artery disease and a low EPA/AA ratio at baseline. This study showed a 21% reduction in major adverse cardiovascular events (hazard ratio, 0.79 [0.62–1.00]) concomitant with significant increases in both EPA levels and the EPA/AA ratio.^30^ Post hoc evaluation of patients in each arm who did (IPE arm) and did not (control arm) achieve increases in EPA levels showed a significant reduction in the primary end point. Together, these data suggest that targeting the EPA/AA ratio as a modifiable risk factor in ASCVD prevention warrants further investigation with IPE. By increasing EPA levels, IPE treatment may favor a balance between pro-inflammatory and pro-resolving oxylipins implicated in atheroma development to augment the other direct cardioprotective mechanisms of EPA.^1^

## ISOLATED OXYLIPINS FOR CLINICAL DEVELOPMENT?

**Figure 2.**
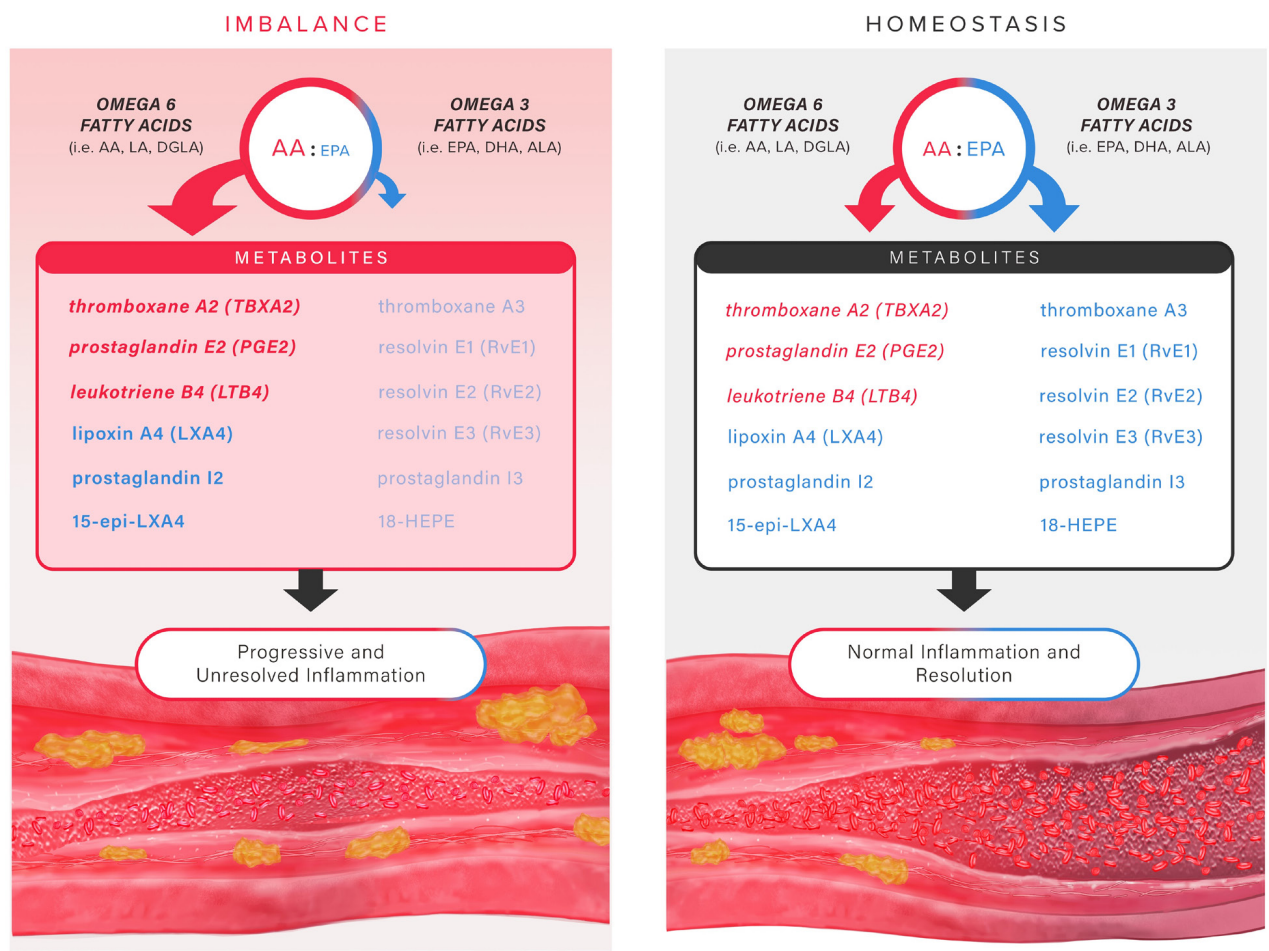
**Insufficient omega-3/omega-6 levels promote inflammation during atherosclerosis in contrast to eicosanoid homeostasis.** When the balance of lipid precursors favors more arachidonic acid (AA) than eicosapentaenoic acid (EPA), there is an insufficient production of pro-resolving eicosanoids. The inflammatory process continues unabated, leading to atherosclerosis. By contrast, when there are adequate levels of both omega-6 fatty acids (especially AA) and omega-3 fatty acids (especially EPA), the inflammatory process initiates and resolves with the temporal production of oxylipins from both omega-6 and omega-3 fatty acids. Pro-inflammatory prostaglandins such as prostaglandin E2 and leukotriene B4 from AA recruit polymorphonuclear leukocytes to extravasate through the endothelium and into the intima to initially clear inflammatory debris. This process yields with the switch in lipid mediators from pro-inflammatory to pro-resolving, which includes the production of lipoxin A4 from AA and resolvins from EPA, docosahexaenoic acid (DHA), and docosapentaenoic acid (DPA), all of which halt polymorphonuclear neutrophil (PMN) infiltration and promote efferocytosis by macrophages. ALA indicates α-linolenic acid; DGLA, dihomo-γ-linolenic acid; and LA, linoleic acid.

These recent cardiovascular outcome trials indicate that balance between n3-PUFAs and n6-PUFAs can alleviate ASCVD risk and events, along with providing pleiotropic benefits associated with therapeutic levels of EPA, in particular. These findings have also inspired new investigations into potential therapeutic actions of isolated oxylipins in various disease settings. As discussed above, these oxylipins may elicit a range of beneficial actions, from reducing inflammatory biomarkers to increasing arterial plaque stability. But we must fully appreciate how these molecules each separately affect such pro-resolving and anti-inflammatory actions to elucidate their role in pathophysiology as well as in the body’s natural homeostatic response mechanisms. Efforts to exploit individual metabolites for therapeutic applications must not come at the expense of restoring the underlying imbalance of the oxylipin precursors, specifically EPA and AA. Indeed, oxylipins are both spatially and temporally generated from these precursors. Circumventing this innate regulation runs the risk of being overly reductionistic by missing the broader mosaic of mechanisms provided by the intact EPA precursor (Table).^1,31^ These mechanisms are precisely regulated to induce and then resolve inflammation as needed in response to injury, and oxylipins participate critically in this process. The most promising path toward sustainably resolving the chronic pro-inflammatory state of arterial lesions unlikely resides in any one oxylipin species. A recent clinical trial with isolated oxylipins, despite some beneficial effects on inflammatory biomarkers, failed to meet its primary end point.^32^ Rather, the most likely magic bullet is one that enables the essential balance of precursor PUFAs needed to disseminate oxylipins as required to restore homeostasis and maintain the normal cycle of tissue breakdown and restoration fundamental to health (Figure 2).

**Table.**
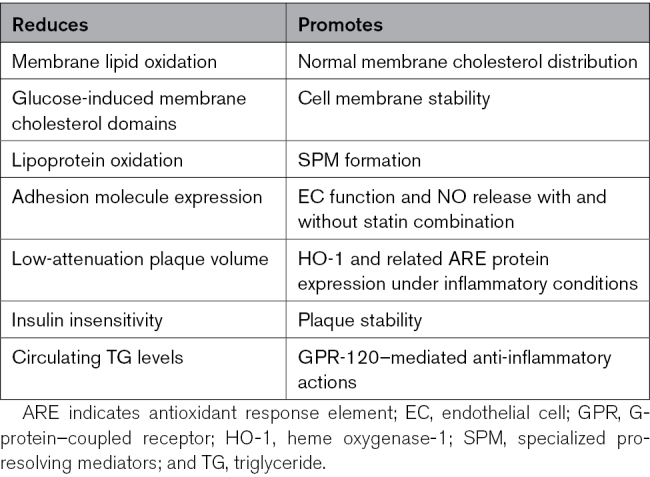
Pleiotropic Actions of Eicosapentaenoic Acid to Reduce Atherosclerosis

## ARTICLE INFORMATION

### Acknowledgments

The authors thank Luke A. Groothoff (Elucida Communications) for creating the figures.

### Sources of Funding

P. Libby receives funding support from the National Heart, Lung, and Blood Institute (1R01HL134892 and 1R01HL163099-01).

### Disclosures

S.C.R. Sherratt is an employee of Elucida Research LLC. R.P. Mason receives research funding from Amarin Pharma, Inc, Lexicon Pharma, Inc, Esperion Therapeutics, and Viatris. P. Libby is an unpaid consultant at, or involved in clinical trials for, Amgen, AstraZeneca, Baim Institute, Beren Therapeutics, Esperion Therapeutics, Genentech, Kancera, Kowa Pharmaceuticals, Medimmune, Merck, Moderna, Novo Nordisk, Novartis, Pfizer, and Sanofi-Regeneron. He is a member of the Scientific Advisory Board for Amgen, Caristo Diagnostics, Cartesian Therapeutics, CSL Behring, DalCor Pharmaceuticals, Dewpoint Therapeutics, Elucid Bioimaging, Kancera, Kowa Pharmaceuticals, Olatec Therapeutics, Medimmune, Novartis, PlaqueTec, TenSixteen Bio, Soley Therapeutics, and XBiotech, Inc. His laboratory has received research funding in the last 2 years from Novartis, Novo Nordisk and Genentech. He is on the Board of Directors of XBiotech, Inc. He has a financial interest in Xbiotech, a company developing therapeutic human antibodies, in TenSixteen Bio, a company targeting somatic mosaicism and clonal hematopoiesis of indeterminate potential to discover and develop novel therapeutics to treat age-related diseases, and in Soley Therapeutics, a biotechnology company that is combining artificial intelligence with molecular and cellular response detection for discovering and developing new drugs, currently focusing on cancer therapeutics. His interests were reviewed and are managed by Brigham and Women’s Hospital and Mass General Brigham in accordance with their conflict-of-interest policies. He Libby receives funding support from the National Heart, Lung, and Blood Institute (1R01HL134892 and 1R01HL163099-01), the RRM Charitable Fund, and the Simard Fund. D.L. Bhatt discloses the following relationships: Advisory Board: Angiowave, Bayer, Boehringer Ingelheim, CellProthera, Cereno Scientific, Elsevier Practice Update Cardiology, High Enroll, Janssen, Level Ex, McKinsey, Medscape Cardiology, Merck, MyoKardia, NirvaMed, Novo Nordisk, PhaseBio, PLx Pharma, Stasys; Board of Directors: American Heart Association New York City, Angiowave (stock options), Bristol Myers Squibb (stock), DRS.LINQ (stock options), High Enroll (stock); consultant: Broadview Ventures, Hims, SFJ, Youngene; Data Monitoring Committees: Acesion Pharma, Assistance Publique-Hôpitaux de Paris, Baim Institute for Clinical Research (formerly Harvard Clinical Research Institute, for the PORTICO trial, funded by St. Jude Medical, now Abbott), Boston Scientific (chair, PEITHO trial), Cleveland Clinic, Contego Medical (chair, PERFORMANCE 2), Duke Clinical Research Institute, Mayo Clinic, Mount Sinai School of Medicine (for the ENVISAGE trial, funded by Daiichi Sankyo; for the ABILITY-DM trial, funded by Concept Medical; for ALLAY-HF, funded by Alleviant Medical), Novartis, Population Health Research Institute; Rutgers University (for the National Institutes of Health–funded MINT trial); honoraria: American College of Cardiology (senior associate editor, Clinical Trials and News, ACC.org; chair, ACC Accreditation Oversight Committee), Arnold and Porter law firm (work related to Sanofi/Bristol Myers Squibb clopidogrel litigation), Baim Institute for Clinical Research (formerly Harvard Clinical Research Institute; RE-DUAL PCI clinical trial steering committee funded by Boehringer Ingelheim; AEGIS-II executive committee funded by CSL Behring), Belvoir Publications (editor in chief, Harvard Heart Letter), Canadian Medical and Surgical Knowledge Translation Research Group (clinical trial steering committees), CSL Behring (AHA lecture), Cowen and Company, Duke Clinical Research Institute (clinical trial steering committees, including for the PRONOUNCE trial, funded by Ferring Pharmaceuticals), HMP Global (editor in chief, *Journal of Invasive Cardiology*), *Journal of the American College of Cardiology* (guest editor; associate editor), K2P (cochair, interdisciplinary curriculum), Level Ex, Medtelligence/ReachMD (CME steering committees), MJH Life Sciences, Oakstone CME (course director, Comprehensive Review of Interventional Cardiology), Piper Sandler, Population Health Research Institute (for the COMPASS operations committee, publications committee, steering committee, and USA national coleader, funded by Bayer), WebMD (CME steering committees), Wiley (steering committee); other: Clinical Cardiology (deputy editor); patent: Sotagliflozin (named on a patent for sotagliflozin assigned to Brigham and Women’s Hospital who assigned to Lexicon; neither he nor Brigham and Women’s Hospital receive any income from this patent); research funding: Abbott, Acesion Pharma, Afimmune, Aker Biomarine, Alnylam, Amarin, Amgen, AstraZeneca, Bayer, Beren, Boehringer Ingelheim, Boston Scientific, Bristol Myers Squibb, Cardax, CellProthera, Cereno Scientific, Chiesi, CinCor, Cleerly, CSL Behring, Eisai, Ethicon, Faraday Pharmaceuticals, Ferring Pharmaceuticals, Forest Laboratories, Fractyl, Garmin, HLS Therapeutics, Idorsia, Ironwood, Ischemix, Janssen, Javelin, Lexicon, Lilly, Medtronic, Merck, Moderna, MyoKardia, NirvaMed, Novartis, Novo Nordisk, Otsuka, Owkin, Pfizer, PhaseBio, PLx Pharma, Recardio, Regeneron, Reid Hoffman Foundation, Roche, Sanofi, Stasys, Synaptic, The Medicines Company, Youngene, 89Bio; royalties: Elsevier (editor, *Braunwald’s Heart Disease*); site coinvestigator: Abbott, Biotronik, Boston Scientific, CSI, Endotronix, St. Jude Medical (now Abbott), Philips, SpectraWAVE, Svelte, Vascular Solutions; trustee: American College of Cardiology; unfunded research: FlowCo.
